# Fully automated identification of brain abnormality from whole-body FDG-PET imaging using deep learning-based brain extraction and statistical parametric mapping

**DOI:** 10.1186/s40658-021-00424-0

**Published:** 2021-11-14

**Authors:** Wonseok Whi, Hongyoon Choi, Jin Chul Paeng, Gi Jeong Cheon, Keon Wook Kang, Dong Soo Lee

**Affiliations:** 1 Department of Molecular Medicine and Biopharmaceutical Sciences, Seoul National University, Seoul, 03080 Republic of Korea; 2 Department of Nuclear Medicine, Seoul National University College of Medicine, 101 Daehak-ro, Jongno-gu, Seoul, 03080 Republic of Korea

**Keywords:** Brain segmentation, Quantitative PET analysis, Deep learning, Convolutional neural network, FDG-PET, Brain FDG-PET

## Abstract

**Background:**

The whole brain is often covered in [^18^F]Fluorodeoxyglucose positron emission tomography ([^18^F]FDG-PET) in oncology patients, but the covered brain abnormality is typically screened by visual interpretation without quantitative analysis in clinical practice. In this study, we aimed to develop a fully automated quantitative interpretation pipeline of brain volume from an oncology PET image.

**Method:**

We retrospectively collected 500 oncologic [^18^F]FDG-PET scans for training and validation of the automated brain extractor. We trained the model for extracting brain volume with two manually drawn bounding boxes on maximal intensity projection images. ResNet-50, a 2-D convolutional neural network (CNN), was used for the model training. The brain volume was automatically extracted using the CNN model and spatially normalized. For validation of the trained model and an application of this automated analytic method, we enrolled 24 subjects with small cell lung cancer (SCLC) and performed voxel-wise two-sample *T* test for automatic detection of metastatic lesions.

**Result:**

The deep learning-based brain extractor successfully identified the existence of whole-brain volume, with an accuracy of 98% for the validation set. The performance of extracting the brain measured by the intersection-over-union of 3-D bounding boxes was 72.9 ± 12.5% for the validation set. As an example of the application to automatically identify brain abnormality, this approach successfully identified the metastatic lesions in three of the four cases of SCLC patients with brain metastasis.

**Conclusion:**

Based on the deep learning-based model, extraction of the brain volume from whole-body PET was successfully performed. We suggest this fully automated approach could be used for the quantitative analysis of brain metabolic patterns to identify abnormalities during clinical interpretation of oncologic PET studies.

## Background

[^18^F]fluorodeoxyglucose positron emission tomography ([^18^F]FDG-PET) has been playing a crucial role in tumor imaging [1, 2]. The clinical implications include diagnosis of unknown tumor, staging, monitoring for recurrence, and clinical assessment of therapy [3]. The routine protocol for oncologic ^18^F-FDG-PET is so-called torso imaging, covering from skull base to mid-thigh in most of the PET centers [4]. However, in practice, covering the whole level of skull is often clinically useful, for example, assessment of brain metastasis in the high-prevalence type of tumor, such as lung cancer [5–7]. The covered brain volume is typically analyzed by visual inspection in clinical practice due to low sensitivity in metastatic lesions compared with magnetic resonance (MR) imaging. Thus, in the clinical practice, roles of oncologic ^18^F-FDG-PET for detection of brain lesions is underestimated [8]. However, incidental brain metastasis is important for clinical decisions. Furthermore, brain metabolism reflecting functional activity is affected by chemotherapy as well as tumors themselves—e.g., paraneoplastic encephalitis, which could affect outcome [9, 10]. As functional image assesses the metabolism of the whole body as well as tumors, quantitative information of covered brain extracted from the oncology FDG-PET study could be utilized to identify brain abnormality as well as unexpected metastasis. More specifically, with the aid of automated analytic methods, such as statistical parametric mapping (SPM) [11, 12], the information from brain PET images might improve sensitivity for detecting incidental brain disorders by measuring regional metabolic abnormalities [13], such as local-onset seizures [14], or Alzheimer’s disease (AD) [15], as well as brain metastasis [16].

Some studies have implemented deep learning-based approaches for automatic analysis of brain images, especially targeting MR imaging. Since MR image typically covers head region only, these studies target segmenting anatomic structures [17] or anatomically apparent lesions [18, 19]. Other studies based on PET images also target segmenting metabolically active tumor lesions [20, 21]. However, no study, as far as we know, has targeted extracting the brain itself from the images covering the whole body.

To achieve this goal, we implemented a convolutional neural network (CNN)-based deep learning model, which has been successful in solving a variety of problems in the field of image processing, including image classification, object detection, and segmentation [22, 23]. A vast portion of this success includes medical image processing [24, 25], including anatomical segmentation of the brain [26] or detection of the tumorous lesion [27] of MR images.

In this study, we aimed to develop a fully automatic quantitative analysis pipeline of brain volume from a given oncology PET image. To achieve this goal, deep learning models were exploited to detect the location of the brain and to identify whether a given PET study included the whole brain. The detected brain was cropped and spatially normalized to the template brain. The automatically extracted and normalized brain volume could be used to perform statistical analysis, including SPM. As an example, we applied this model to identify brain metastasis from whole-body FDG-PET imaging.

## Methods

### Subjects

For the training and validation data of the automatic brain extractor, 500 whole-body [^18^F]FDG-PET scans were retrospectively collected. These PET scans were performed from June to July 2020 in a single center (Age = 66.7 ± 3.4, M: F = 194: 306). The scans which were explicitly prescribed to include the brain (by the oncologic clinicians) were excluded from analysis, to remove the bias in the evaluation of detecting accuracy of brain existence. Among the 500 cases, the primary site of malignancy was breast (19.8%), lung (18.6%), hematologic (14.2%), colorectal (9.4%), biliary (6.8%), ovary (5.4%), pancreas (5.2%), liver (5.0%), stomach (4.2%), thymus (3.6%), urinary tract (3.4%), soft tissue (3.0%), thyroid (0.6%), or unknown (0.8%).

For the validation of our trained model and the quantitative assessment of the extracted brain as an independent test, FDG-PET images acquired from small-cell lung cancer (SCLC) patients were retrospectively collected. The scans were acquired from January 2014 to December 2017 in the same institute. To test whether our automated brain analysis pipeline identifies brain metastasis in SCLC patients, groups were defined according to the presence of brain metastasis. Four patients had brain metastasis confirmed by brain MRI at baseline and follow-up (age: 66.8 ± 6.5, M: F = 4: 0). Twenty PET scans without brain metastasis, according to the baseline brain MRI were regarded as controls (age: 71.2 ± 6.1; M: F = 17:3).

### Image acquisition

As a routine protocol of FDG-PET, after fasting more than 4 h, patients were intravenously injected with 5.18 MBq/kg of FDG. After 1 h, PET image was acquired from the skull base to the proximal thigh using dedicated PET/CT scanners (Biograph mCT 40 or mCT 64, Siemens, Erlangen, Germany) for 1 min per bed. A Gaussian filter (FWHM 5 mm) was applied to reduce noise, and images were reconstructed using an ordered-subset expectation maximization algorithm (2 iterations and 21 subsets).

### Deep learning model and training data for the brain extraction

**Fig. 1:**
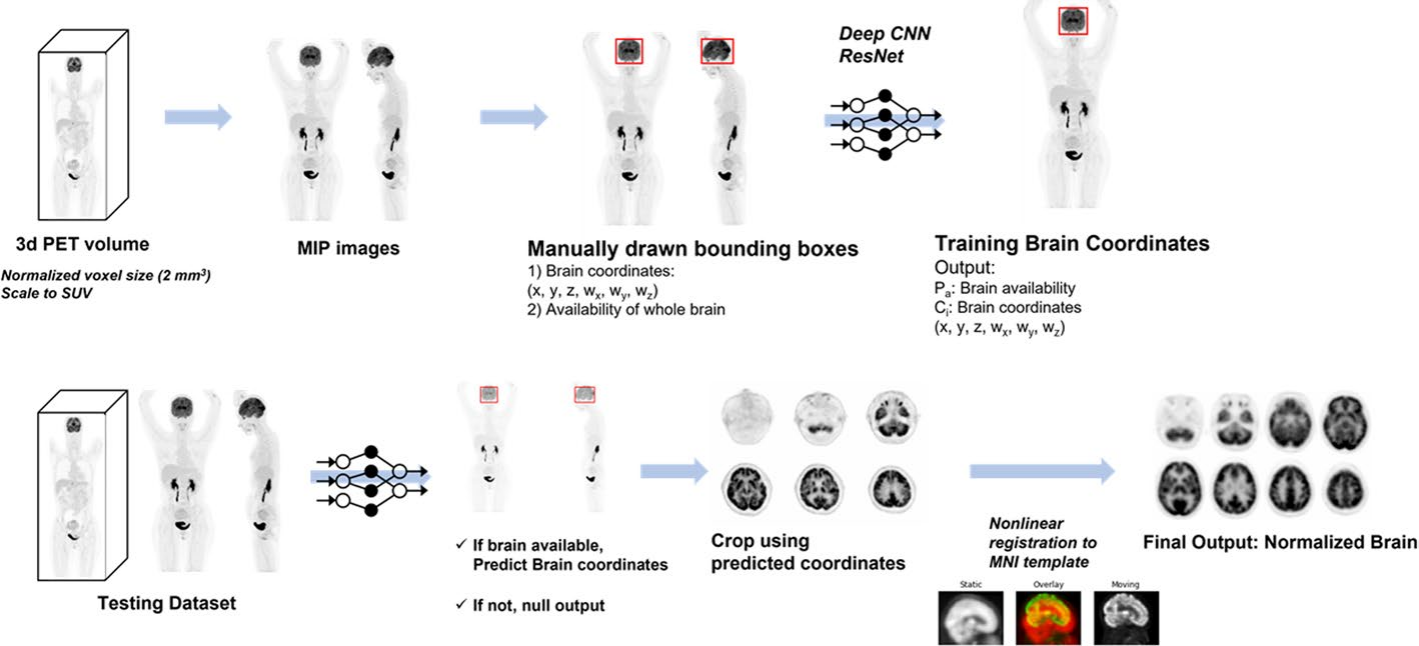
Brief outline of the automatic brain extraction. We trained the model with two manually drawn bounding boxes on maximal intensity projection (MIP) images. ResNet-50, a convolutional neural network (CNN) was used for learning model. Internal validation of model was performed. Finally, the brain volume was extracted and spatially normalized to the template space

We devised an automatic brain extractor based on the following two objectives: (1) the evaluation of whether a scan included the entire brain and (2) the establishment of a 3-D bounding box which included brain volume. The brief outline of the study is shown in Fig. 1.

For training of the model, the maximum intensity projection (MIP) image for each of the PET scans was generated. For each of the 500 MIP images, 2-D bounding boxes were manually drawn on the anterior and lateral views of the MIP image. We used VGG Image Annotator (VIA) [28] to manually create bounding boxes on the MIP images and acquire coordinates of them. Coordinates from the two bounding boxes were merged to obtain coordinates of a 3-D bounding box for each PET image. Images that did not contain full range of the brain were classified elsewhere, as “not containing entire brain”.

Two MIP images, anterior and lateral views, were changed to square matrices by zero-padding. The matrices were changed to 224 × 224 using bilinear interpolation. The pixel values represented the standardized uptake value (SUV). To be inputs of a CNN model, pixel values were divided by 30, as most voxel values of PET volume have less than SUV 30 except urine, and then multiplied by 255 to have a range approximately 0 to 255.

We utilized ResNet-50 [29, 30] for the learning model, a 2-D CNN pre-trained with images from the ImageNet database [31]. The module used the Python front end of the open-source library TensorFlow [32], which runs on Graphical Processing Unit (GPU, NVIDIA GeForce RTX 2080Ti). ResNet-50 was implemented for preprocessing the input data and predicting the coordinates of 3-D bounding boxes from the MIP images. The pre-trained ResNet-50 respectively extracted feature vectors from the two views of MIP images. The extracted features were concatenated. An additional fully connected layer with 4096 dimensions was connected to the concatenated feature vectors and then finally connected to different outputs. An output represented coordinates of the bounding box of the brain consisting of 6-D vectors (coordinates for three axes and width, length, and depth of the bounding box for three axes). Another output with a 1-D vector represented whether a given PET volume included the entire brain. Image augmentation was applied to the training dataset. MIP images were randomly augmented by multiplying voxel values, changing contrast, scaling, and translating images. For the optimizer, we implemented *Adam* [33] with a learning rate of 0.00001, 150 epochs, and batch size of 8.

We performed the internal validation by randomly selecting 10% of the data (*n* = 50) as a validation set. The loss function was defined by two terms:

$$\begin{aligned} & L_{a} = Y_{i} \log \left( {p\left( y \right)} \right) + \left( {1 - Y_{i} } \right)\log \left( {1 - p\left( y \right)} \right) \\ & L_{b} = Y_{i} \mathop \sum \limits_{{j = x, y,z,w_{x} ,w_{y} ,w_{z} }} (y_{b,j} - \hat{y}_{b,j} )^{2} \\ & L_{{\text{all }}} = \alpha L_{a} + \beta L_{b} \\ \end{aligned}$$

where ${Y}_{i}$ indicates the true existence of the brain (equal to 1 when the whole brain exists, 0 when not), $p\left(y\right)$ denotes the predicted existence, and vectors $y$ and $\widehat{y}$ denote the predicted and true coordinates of the bounding box, respectively. Therefore, two terms of the loss function, ${L}_{a}$ and ${L}_{b}$ represent (1) binary cross-entropy of an output that represented whether a given PET volume included the entire brain and (2) mean squared error estimated by the 6-D vector representing coordinates of the bounding box, respectively (Fig. 1). The weight for the loss was empirically determined for the training: we set to alpha = 10 and beta = 1 for sum of the loss function. We measured intersection-over-union (IOU) for the predicted and labeled bounding boxes. From the predicted coordinates of bounding boxes, we extracted brain images from whole-body PET and spatially normalized them to the template space, as mentioned later.

### Processing of the extracted brain

The trained model was applied to whole-body PET images to extract brain if the model predicted that the image contains the whole-brain volume. FDG-PET volumes were resliced to have a voxel size of 2 × 2 × 2 mm^3^. We segmented the brain with the coordinates of the 3-D bounding boxes predicted by the model. Padding of 10 voxels is applied for each axis to determine the brain volume. The extracted brain volumes were spatially normalized onto Montreal Neurological Institute (McGill University, Montreal, Quebec, Canada) standard templates. The spatial normalization was performed by symmetric normalization (SyN) with the cross-correlation loss function implanted in the DIPY package [34]. More specifically, a given extracted brain volume was linearly transformed to the template PET image with affine transform. The warping was performed by the symmetric diffeomorphic registration algorithm. The spatially normalized PET volume was saved for further quantitative imaging analysis.

### Quantitative analysis of the extracted brain

The extracted and spatially normalized brain volume was analyzed by a quantitative software, SPM12 (Institute of Neurology, University College of London, London, U K) implemented in MATLAB 2019b (The MathWorks, Inc., Natick, MA, U SA). The normalized brain images were smoothed by convolution with an isotropic Gaussian kernel having a 10 mm full width at half maximum to increase the signal-to-noise ratio.

For the 24 subjects with SCLC, we performed the voxel-wise two-sample T test for each of the normalized brain volumes from the four scans with metastatic lesions, with the whole images from the 20 control group subjects. Uncorrected *P* < 0.001 was applied to identify patient-wise metabolically abnormal regions.

For each of the four comparisons, we also constructed a map of *T*-statistics and extracted the peak *T* values. As a proof-of-concept study, we investigated whether the statistical analysis successfully revealed the metastatic lesions confirmed by the brain MRI previously.

## Results

### Extraction of the brain volume

The deep learning-based brain extractor successfully identified the existence of whole-brain volume, with an accuracy of 98% for the internal validation set. The performance of extracting the brain measured by the IOU of 3-D bounding boxes was 72.9 ± 12.5% for the validation set. Using the predicted coordinates, all brains were successfully cropped and automatically normalized into the template space.

**Fig. 2:**
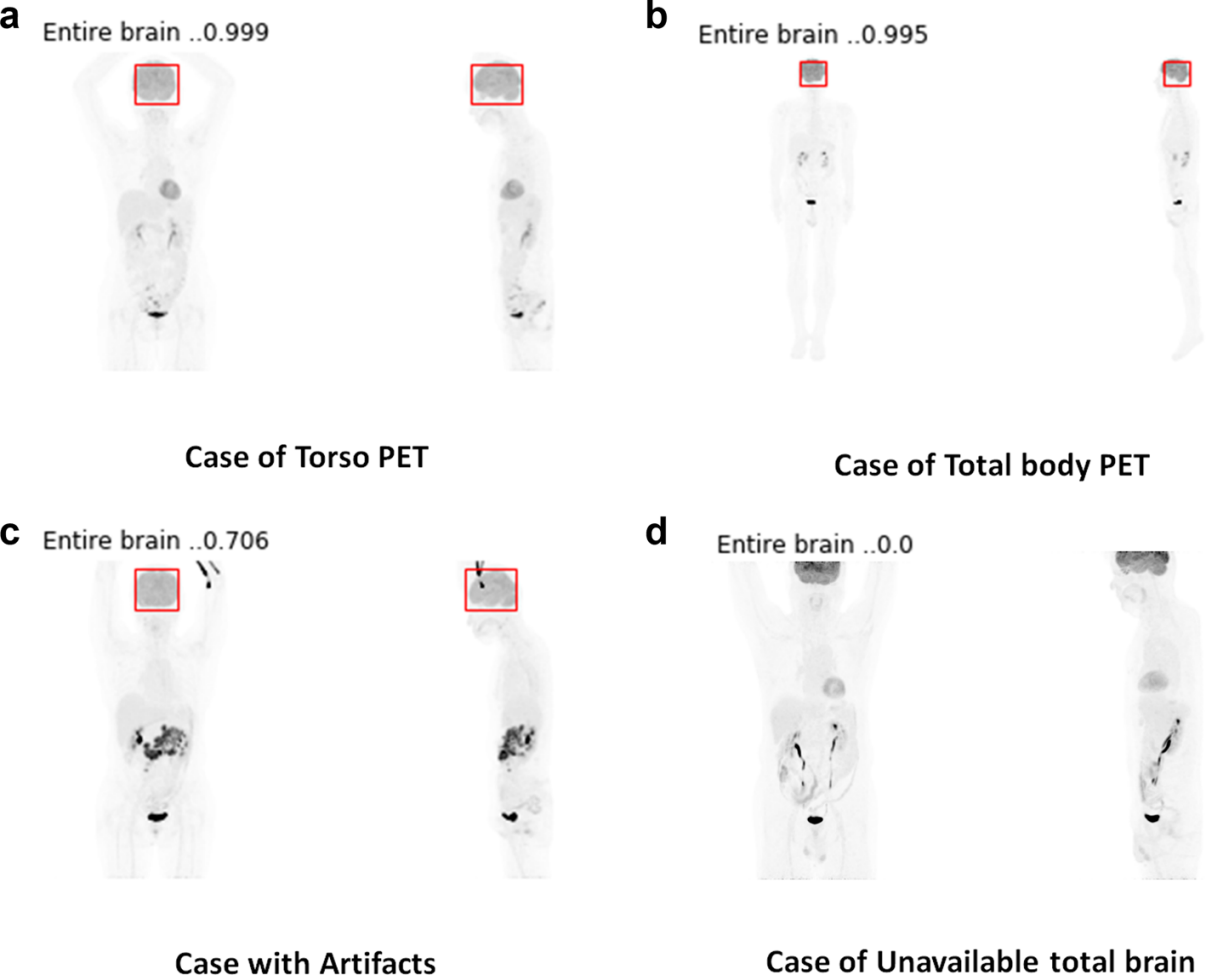
Representative results of the automatic brain extractor. **a**, **b** In both of the “torso”, PET covering up to mid-thigh and “total-body” PET covering whole heights of body the extractor successfully located the brain. **c** The extractor was also capable of identifying brain when the artifact caused by radiopharmaceutical injection was projected to the brain at the MIP image. **d** When the brain volume was not fully included, the extractor classified the image as “not containing entire brain”

We show some representative images we applied for interval validation of the model in Fig. 2. In both of the “torso” PET covering up to mid-thigh and “total-body” PET covering whole heights of the body the extractor successfully located the brain (Fig. 2a, b). The extractor was also capable of identification of the brain when the artifact caused by radiopharmaceutical injection was projected to the brain at the MIP image (Fig. 2c). When the brain volume was not fully included, the extractor classified the image as “not containing entire brain” (Fig. 2d).

### Identification of brain metastasis from whole-body FDG-PET using the fully automated brain analysis pipeline

**Fig. 3:**
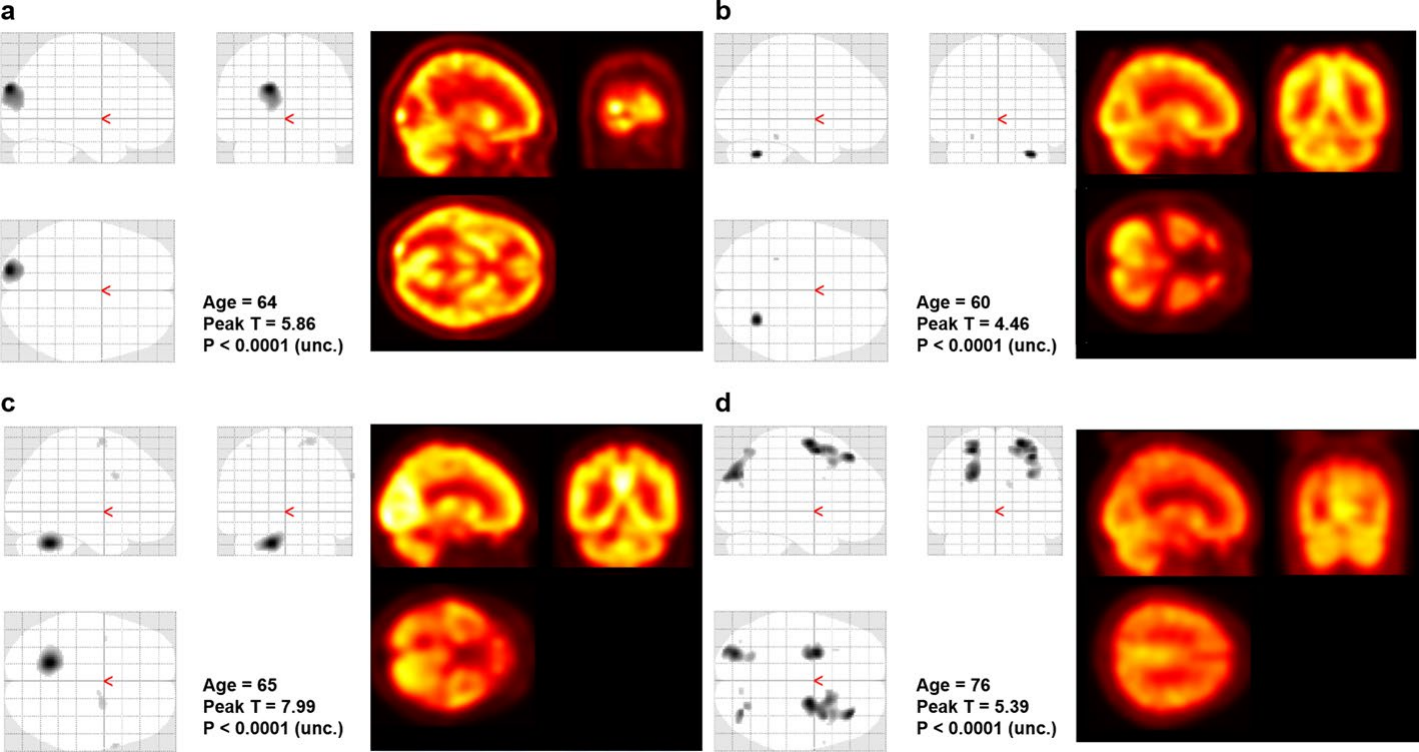
Quantitative analysis of the extracted brain. The voxel-wise T test successfully identified the metastatic lesions in the brain at three of four subjects in the case group (uncorrected *P* < 0.001). The graphics on the left side show the brain regions that show hypometabolism compared to the control group. The image on the right side shows the corresponding FDG-PET image. **a**, **b**, **c** In all of the three successful cases, the analysis revealed hypometabolic lesions due to edematous change around the lesion. **d** In the other case with unsuccessful result, the statistical analysis showed diffuse hypometabolism in frontoparietal lobe, instead of focal metabolic defect at the metastatic site

The fully automated brain extraction and quantitative analysis were applied to the patients with SCLC as a proof-of-concept study. In the 24 whole-body PET images, the automatic brain extractor has identified the existence of the whole brain with an accuracy of 100%, and the IOU of 3-D bounding boxes was 75.8 ± 7.2%. The voxel-wise T test successfully identified the metastatic lesions in the brain at three of four subjects in the case group (*P* < 0.001). In all of the three successful cases, the analysis revealed hypometabolic lesions due to edematous change around the lesion (Fig. 3). In the other case with the unsuccessful result, the statistical analysis showed diffuse hypometabolism in the frontoparietal lobe, instead of focal metabolic defect at the metastatic site (Fig. 3).

## Discussion

Since the increased utilization of FDG-PET in neurologic disorders, many kinds of literature suggest methods for quantitative analysis for FDG-PET images of the brain [12, 35, 36]. However, most of the subjects with FDG-PET scans, especially oncologic patients, are not benefited from this kind of progress, for the lack of a handy and automated method of quantification. This work aims to achieve the first step of this automatization by deep learning-based extraction of brain volume from the oncologic PET scan, which is followed by a scout quantitative analysis of the extracted brains. Fully automated brain extraction and providing quantitative information in oncologic PET can be integrated into a system that warns of metastatic lesions or major brain diseases that can be overlooked in visual reading.

The key step of automated quantitative brain imaging analysis from the oncologic PET images was the extraction of brain volumes. In most of the scans of internal validation, the automatic brain extractor based on ResNet-50 successfully identified the coverage of full brain in the whole-body scan and located and extracted the brain volume, even in the presence of artifact projected to the MIP image.

The extracted brain PET volume can be analyzed by many conventional quantitative analysis approaches. In this study, for the fully automated process, we employed a spatial normalization process based on SyN algorithm implemented in the DIPY package. Notably, the spatial normalization process after the brain extraction was fully automated. The spatially normalized brain can be further analyzed by quantitative software, including SPM and 3-D stereotactic surface projection (3-D SSP) [37]. In this work, as a proof-of-concept study, we implemented SPM to identify metastatic lesions. This reveals the implication of the automatic brain extraction we performed, which could potentially extend to aid in the identification of unexpected metastasis during visual interpretation of oncologic PET study. Moreover, this method could be used to identify overlooked brain abnormalities such as dementia as well as tumorous lesions in the brain.

In the process of the quantitative analysis, as a proof-of-concept study, age matching was not performed between the metastatic subject and control group to yield rather non-specific decreased metabolism along the cerebral cortex in an elderly subject. This might have resulted from the physiologic decrease in gray matter volume accompanied by a normal aging process [38, 39]. Adjustment of patient factors (e.g., age and underlying disease) would be crucial to detect a localized metabolic disorder, apart from the diffuse change of metabolism resulted from the systemic condition. In addition, although the brain extraction model showed good results, there is room for optimization such as hyperparameter tuning and revising model architecture. Nonetheless, considering that the purpose of the model was ‘spatially normalized brain’, which could be obtained from the extracted brain even with small errors in the brain coordinates. As a proof-of-concept study, our suggested model has proved the final purpose of identifying brain abnormality from the automatically spatially normalized brain.

## Conclusions

Based on the deep learning-based model, we successfully developed a fully automated brain analysis method from oncologic FDG-PET. The model could identify the existence of the brain volume, locate the contour of brain from the PET image, and perform the spatial normalization to the template. The quantitative analysis showed the feasibility of the identification of the metastatic brain lesion. We suggested that the model could be used to support FDG-PET interpretation and analysis by finding unexpected brain abnormalities, including metastasis as well as brain disorders.

## Data Availability

Not applicable.

## Abbreviations

FDG: Fluorodeoxyglucose
PET: Positron emission tomography
MIP: Maximal intensity projection
CNN: Convolutional neural network
SCLC: Small-cell lung cancer
IOU: Intersection-over-union
MR: Magnetic resonance
SPM: Statistical parametric mapping
AD: Alzheimer’s disease
SUV: Standardized uptake value
SyN: Symmetric normalization
SSP: Stereotactic surface projection
